# *Malpais spring virus* is a new species in the genus *vesiculovirus*

**DOI:** 10.1186/1743-422X-10-69

**Published:** 2013-03-04

**Authors:** Nikos Vasilakis, Steven Widen, Amelia PA Travassos da Rosa, Thomas G Wood, Peter J Walker, Edward C Holmes, Robert B Tesh

**Affiliations:** 1 Center for Biodefense and Emerging Infectious Diseases and Department of Pathology, University of Texas Medical Branch, Galveston 77555-0609, TX, USA; 2 Center for Tropical Diseases, University of Texas Medical Branch, Galveston 77555-0609, TX, USA; 3 Institute for Human Infections and Immunity, University of Texas Medical Branch, 77555-0610, Galveston, TX, USA; 4 Department of Biochemistry and Molecular Biology, University of Texas Medical Branch, 77555-0679, Galveston, TX, USA; 5 CSIRO Animal, Food and Health Sciences, Australian Animal Health Laboratory, VIC 3220, Geelong, Australia; 6 Sydney Emerging Infections & Biosecurity Institute, School of Biological Sciences and Sydney Medical School, The University of Sydney, 2006, Sydney, NSW, Australia; 7 Fogarty International Center, National Institutes of Health, Bethesda, Maryland, USA

**Keywords:** Malpais spring virus (MSPV), Family *rhabdoviridae*, Genus *vesiculovirus*, Genome sequence, Phylogeny

## Abstract

**Background:**

Malpais Spring virus (MSPV) is a mosquito-borne rhabdovirus that infects a variety of wild and feral ungulates in New Mexico, including horses and deer. Although, initial serologic tests and electron microscopy at the time of isolation nearly 25 years ago provided evidence that MSPV is a novel virus, possibly related to vesiculoviruses, the virus still has not been approved as a new species.

**Findings:**

Use of the illumina platform allowed us to obtain the complete genome of MSPV. Analysis of the complete 11019 nt genome sequence of the prototype 85-488NM strain of MSPV indicates that it encodes the five common rhabdovirus structural proteins (N, P, M, G and L) with alternative ORFs (> 180 nt) in the N, M and G genes, including a 249 nt ORF in the G gene predicted to encode a 9.26 kDa highly basic transmembrane protein. Although antigenically very distant, phylogenetic analysis of the L gene indicates that MSPV is most closely related to Jurona virus, also isolated from mosquitoes in Brazil, as well as a number of other vesiculoviruses.

**Conclusions:**

In sum, our analysis indicates MSPV should be classified as a member of the genus *Vesiculovirus*, family *Rhabdoviridae*. The complete genome sequence of MSPV will be helpful in the development of a reverse genetics system to study the unique aspects of this vesiculovirus *in vivo* and *in vitro*, and will assist development of specific diagnostic tests to study the epidemiology of MSPV infection.

## Background

Malpais Spring virus (MSPV) is a negative-sense RNA virus currently assigned to the family *Rhabdoviridae* in the order *Mononegavirales*[1]. MSPV was first isolated from pools of *Aedes campestris* and *Psorophora signipennis* mosquitoes collected during an investigation of unexplained deaths among feral horses living near a spring in the Malpais Lava Flow on the White Sands Missile Range in New Mexico, USA [2]. Initial serologic tests and electron microscopy provided evidence that MSPV is a novel rhabdovirus, possibly related to vesiculoviruses [2]. MSPV is lethal to newborn mice following intracerebral (i.c.) inoculation and causes a cytopathic effect (CPE) in Vero cells, forming sizeable (3–4 mm in diameter) plaques six days post-inoculation [2]. Plaque reduction neutralization tests conducted using sera from wild mammals living in the area where the virus was recovered, indicated a relatively high prevalence (30-100%) of specific MSPV neutralizing antibodies among feral horses, coyotes, mule deer, gemsbok (*Oryx gazella*) and pronghorn, although the virus could not be clearly associated with the horse deaths [2].

## Findings

### Methodology

#### Virus source

The prototype strain of MSPV (85-488NM) was obtained from the World Reference Center for Emerging Viruses and Arboviruses at the University of Texas Medical Branch. The virus stock used had been passaged four times by i.c. inoculation of newborn mice and three times in Vero cells.

#### Next generation sequencing

The complete genome of MSPV (85-488NM) was determined by *de novo* sequencing using the Illumina platform.

##### Library construction

Viral RNA (0.05-1.7 μg) was fragmented by incubation at 94^o^ C for eight (8) minutes in 19.5 μl of fragmentation buffer (Illumina 15016648). First and second strand synthesis, adapter ligation and amplification of the library were performed using the Illumina TruSeq RNA Samplec Preparation kit under conditions prescribed by the manufacturer (Illumina). Samples were tracked using the “index tags” incorporated into the adapters as defined by the manufacturer.

##### Sequence analysis

Cluster formation of the library DNA templates was performed using the TruSeq PE Cluster Kit v3 (Illumina) and the Illumina cBot workstation using conditions recommended by the manufacturer. Paired end 50 base sequencing by synthesis was performed using TruSeq SBS kit v3 (Illumina) on an Illumina HiSeq 1000 using protocols defined by the manufacturer. Cluster density per lane was 645–980 k/mm^2^ and post filter reads ranged from 148–178 million per lane. Base call conversion to sequence reads was performed using CASAVA-1.8.2. Virus assembly was performed using SeqMan Lasergene software (DNASTAR). In certain cases, pre-filtering of reads to remove host sequence enhanced the assembly process. Assembly was carried out using a fasta file of hamster ribosomal RNA sequences to remove host DNA from the assembly, thus reducing the number of contigs present.

#### Nucleotide sequence accession number

The GenBank accession number of MSPV prototype 85-488NM strain is KC412247.

#### Phylogenetic analyses

The L protein sequence of MSPV was compared with those of 44 other rhabdoviruses downloaded from GenBank (members of the genera *Cytorhabdovirus*, *Novirhabdovirus* and *Nucleorhabdovirus* were excluded because their excessive divergence reduced phylogenetic resolution). All protein sequences were aligned using MUSCLE [3] under default settings, and ambiguously aligned regions were then removed using the Gblocks program [4]. This resulted in a final sequence alignment of 1136 amino acid residues. The phylogenetic relationships among these sequences were determined using the maximum likelihood (ML) method available in PhyML 3.0 [5], employing the WAG+Γ model of amino acid substitution and subtree pruning and regrafting (SPR) branch-swapping. The robustness of each node was determined using 1,000 bootstrap replicates utilizing nearest neighbor interchange (NNI) branch-swapping.

#### Antigens and immune reagents

Antigens used in complement fixation (CF) tests and for immunizing animals were prepared from infected newborn mouse brains by the sucrose/acetone extraction method [6]. Hyperimmune mouse ascitic fluids were prepared against each of the 21 rhabdoviruses listed in Table 1. The immunization schedule consisted of four intraperitoneal injections given at weekly intervals. Immunogens consisted of 10% crude suspensions of homogenized infected mouse brain mixed with equal volumes of complete Freund's adjuvant just prior to inoculation. Sarcoma 180 cells were also given intraperitoneally after the final immunization in order to induce ascites formation. All animal experiments were carried out under an animal protocol approved by the University of Texas Medical Branch IACUC committee.

**Table 1:** **Antigenic relationships of MSPV with confirmed and tentative members of the genus *Vesiculovirus***

**Genus**	**Viral antigen**	MSPV antibody*	**Homologous antibody**
	Carajas (CJSV)	0	128
	Chandipura (CHPV)	0	256
	Cocal (COCV)	0	256
	Isfahan (ISFV)	0	64
***Vesiculovirus***	Maraba (MARV)	0	512
	Piry (PIRV)	0	64
	Moreton (MORV)	0	256
	VSV Indiana (VSIV)	0	256
	VSV New Jersey (VSNJV)	0	32
	Boteke (BTKV)	0	256
	Farmington (FARV)	0	256
	Gray Lodge (GLOV)	0	8
	Jurona (JURV)	0	1024
**Tentative**	Klamath (KLAV)	0	256
***Vesiculovirus***	Kwatta (KWAV)	0	64
	La Joya (LJV)	0	512
	Perinet (PERV)	0	512
	Porton (PORV)	0	256
	Radi (RADV)	0	512
	Jug Bogdanovac (YBV)	0	256
	Malpais Spring (MSPV)	512	-

* Antibody titers expressed as reciprocal of highest positive antibody dilution. 0 = <8.

#### Serologic testing

Complement fixation tests were conducted according to a microtechnique described previously [7], using 2 full units of guinea-pig complement. Titers were recorded as the highest dilutions giving 3+ or 4+ fixation of complement on a scale of 0–4+.

## Results

**Figure 1:**
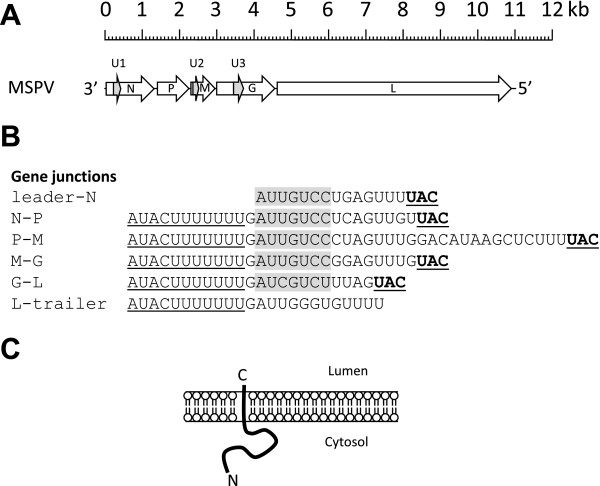
**Genome organization of MSPV. A**. Schematic representation of the genome organization of MSPV. Block arrows indicate the location of long ORFs, including overlapping ORFs in the N, M and G genes (light shading). **B**. Sequences of MSPV gene junctions illustrating potential transcription initiation sequences (shaded), conserved transcription termination/polyadenylation sequences (solid underlined) and initiation codons (dotted underline). **C**. Schematic representation of the predicted membrane topology of a small, basic transmembrane protein encoded in the alternative ORF in the G gene.

The complete genome of MSPV (85-488NM) showed that the 11,019 nt MSPV genome exhibits typical rhabdovirus organization and consists of 3’- and 5’- terminal non-coding regions (approximately 63 nt and 97 nt, respectively) and 5 coding regions (genes), each bounded by putative transcriptional regulatory sequences (Figure 1A). Similar to those of most other animal rhabdoviruses, the transcription initiation (AU[U/C]GUC) signal and transcription termination/polyadenylation ([G/U]UAC[U]_7_) signal are relatively conserved (Figure 1B). Gene 1 (N), encodes a 426-aa protein (47.4 kDa); gene 2 (P), encodes a 274-aa acidic protein (29.8 kDa; pI 4.75); gene 3 (M), encodes a 226-aa protein (25.6 kDa); gene 4 (G), encodes a 522-aa protein, which is a class 1 transmembrane glycoprotein; and gene 5 (L), encodes a 2096-aa protein (238.7 kDa), which the RNA-dependent RNA polymerase (RdRp). Alternative ORFs (< 180 nt) also occur in the N gene (198 nt), M gene (189 nt) and G gene (249 nt) encoding potential polypeptides for which BlastP searches revealed no significant sequence relationships to known proteins (Figure 1A). The putative proteins encoded in the N and M genes are unremarkable in structure. The putative 83-aa product encoded in the G gene is predicted (Phobius and HMMTOP) to be a highly basic transmembrane protein (9.26 kDa; pI 11.36) with a 53-aa N-terminal cytoplasmic domain, a 24-aa helical transmembrane domain and a very short (6-aa) C-terminal ectodomain (Figure 1C). ORFs encoding similar small hydrophobic proteins of unknown function have been identified in the genomes of other rhabdoviruses [8]. The MSPV G protein contains 12 cysteine residues in the ectodomain that form six disulphide bridges that are common to all vesiculovirus G proteins and three predicted N-glycosylation sites, two of which align with those of the vesicular stomatitis Indiana virus (VSIV) G protein [9].

Serologic studies conducted at the time of MSPV isolation indicated a weak antigenic relationship with another probable vesiculovirus Jurona virus (JURV) by indirect immunofluorescence, but none with VSIV, vesicular stomatitis New Jersey, Cocal, Piry or Chandipura viruses (CHPV) [2]. We could not demonstrate an antigenic relationship in CF tests between MSPV and an expanded set of confirmed and tentative vesiculovirus species, including JURV (Table 1).

**Figure 2:**
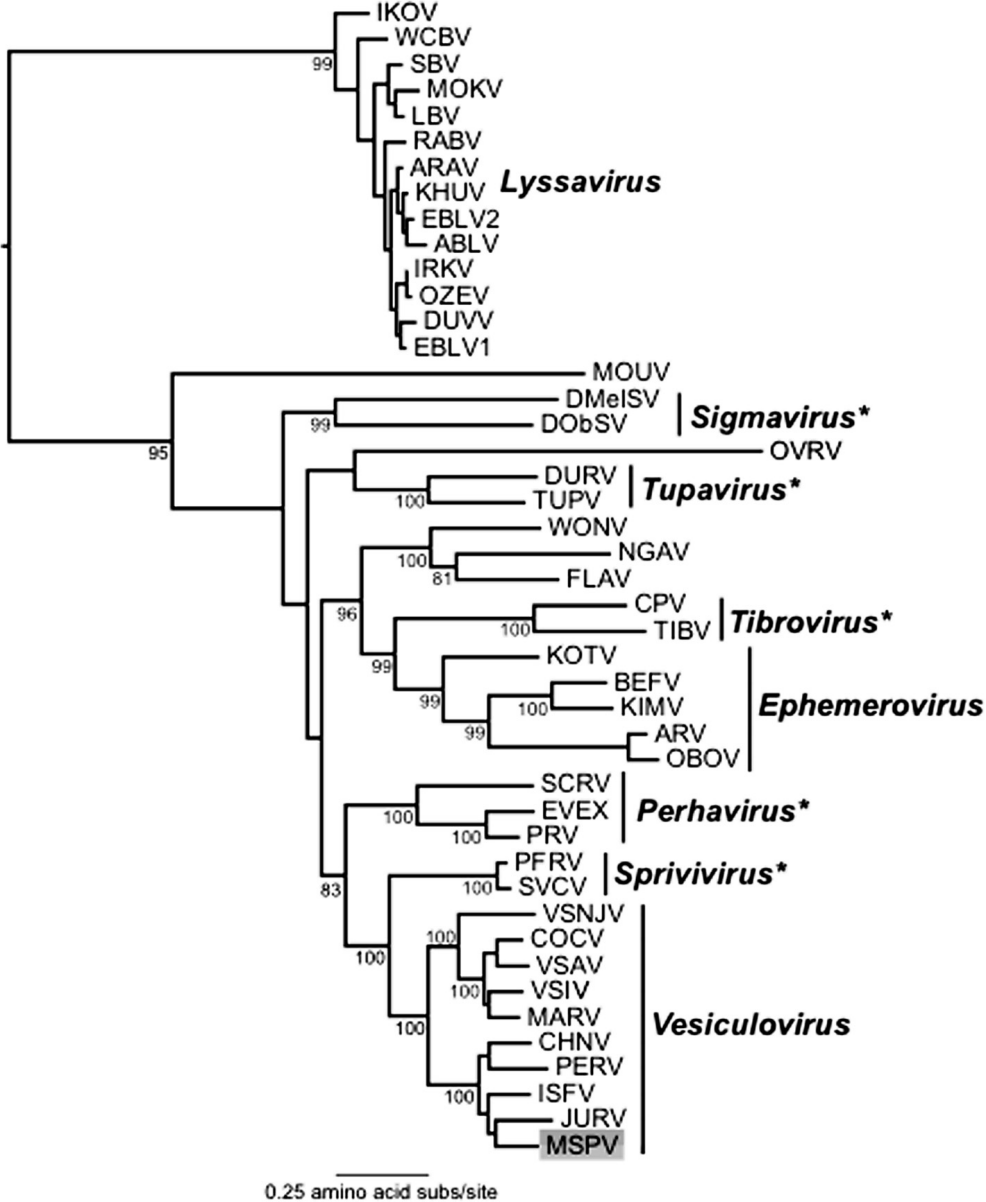
**Phylogenetic tree of L protein sequences from 45 rhabodoviruses.** The position of MSPV is shaded and bootstrap support values (>70%) are shown for key nodes. All horizonal branch lengths are drawn to a scale of amino acid substitutions per site, and the tree is rooted in the position observed in a broader analysis of the *Rhabdoviridae* (i.e. including members of the *Cytorhabdovirus*, *Novirhabdovirus* and *Nucleorhabdovirus*; not shown). (*) Genera not formally approved by the International Committee on Taxonomy of Viruses (ICTV).

Amino acid sequence alignments (MUSCLE) using available rhabdovirus N, G and L proteins indicated that MSPV shares highest sequence identity to vesiculoviruses. Phylogenetic analysis based on an alignment of 1136 amino acid residues of the L protein sequence of 45 rhabdoviruses indicated that MSPV is most closely related (although with weak bootstrap support) to JURV, which has been isolated from *Haemagogus* spp. mosquitoes in the state of Para, Brazil in 1962 [10,11], as well as to Isfahan, CHPV and Perinet viruses (Figure 2). These five viruses form a well-supported (100% bootstrap support) phylogenetic cluster within the genus *Vesiculovirus*.

## Discussion

The genome organization and phylogenetic relationships indicate that Malpais Spring virus should be classified as a new species in the genus *Vesiculovirus.* This is also consistent with the previous report of a distant antigenic relationship with JURV, which also falls phylogenetically within the vesiculovirus cluster. Evidence of alternative ORFs in the MSPV N, M and G genes is unique amongst vesiculovirus genomes sequenced to date but overlapping ORFs appear to occur commonly in rhabdoviruses [8], including VSIV in which an alternative ORF in the P gene encodes the 55-aa and 65-aa C and C’ proteins which are known to be expressed in infected cells from alternative initiation codons [12,13]. The complete genome sequence of MSPV will be helpful in the development of a reverse genetics system to study the unique aspects of this vesiculovirus *in vivo* and *in vitro*, and will assist development of specific diagnostic tests to study the epidemiology of MSPV infection.

## Abbreviations

MSPV: Malpais Spring virus; CPE: Cytopathic effect; i.c: Intracerebral; ML: Maximum likelihood; NNI: Nearest neighbor interchange; CF: Complement fixation; RdRp: RNA dependent RNA polymerase; VSIV: Vesicular stomatitis Indiana virus; JURV: Jurona virus; CHPV: Chandipura virus

## Competing interests

The authors do hereby declare that they have no competing interests in this scientific work.

## Authors’ contributions

NV, SW, TG, RBT performed the laboratory experiments. APATdR performed the serologic assays. ECH performed the phylogenetic analyses; PJW performed the genomic analyses. NV, ECH, PJW, RBT contributed in finalizing the manuscript. All authors have read and approved the final manuscript.

## References

[B1] Dietzgen RG Calisher CH Kurath G Kuzmin IV Rodriguez LL Stone DM Tesh RB Tordo N Walker PJ Wetzel T Whitfield AE King AMQ, Adams MJ, Carstens EB, Lefkowitz EJ Rhabdoviridae Virus Taxonomy, Ninth Report of the International Committe on Taxonomy of Viruses 2012 London: Elsevier/Academic Press 686 713

[B2] Clark GG Calisher CH Crabbs CL Canestorp KM Tesh RB Bowen RA Taylor DE Malpais spring virus: a new vesiculovirus from mosquitoes collected in New Mexico and evidence of infected indigenous and exotic ungulates Am J Trop Med Hyg 1988 39 586 592 3061310 10.4269/ajtmh.1988.39.586

[B3] Edgar RC MUSCLE: a multiple sequence alignment method with reduced time and space complexity BMC Bioinforma 2004 5 113 10.1186/1471-2105-5-113 PMC517706 15318951

[B4] Talavera G Castresana J Improvement of phylogenies after removing divergent and ambiguously aligned blocks from protein sequence alignments Syst Biol 2007 56 564 577 10.1080/10635150701472164 17654362

[B5] Guindon S Dufayard JF Lefort V Anisimova M Hordijk W Gascuel O New algorithms and methods to estimate maximum-likelihood phylogenies: assessing the performance of PhyML 3.0 Syst Biol 2010 59 307 321 10.1093/sysbio/syq010 20525638

[B6] Clarke DH Casals J Techniques for hemagglutination and hemagglutination-inhibition with arthropod-borne viruses Am J Trop Med Hyg 1958 7 561 573 13571577 10.4269/ajtmh.1958.7.561

[B7] Tesh RB Travassos Da Rosa AP Travassos Da Rosa JS Antigenic relationship among rhabdoviruses infecting terrestrial vertebrates J Gen Virol 1983 64 169 176 10.1099/0022-1317-64-1-169 6337233

[B8] Walker PJ Dietzgen RG Joubert DA Blasdell KR Rhabdovirus accessory genes Virus Res 2011 162 110 125 10.1016/j.virusres.2011.09.004 21933691 PMC7114375

[B9] Walker PJ Kongsuwan K Deduced structural model for animal rhabdovirus glycoproteins J Gen Virol 1999 80 1211 1220 10355768 10.1099/0022-1317-80-5-1211

[B10] Karabatsos N Jurona virus International catalogue of arboviruses including certain other viruses of vertebrates 1985 3 San Antonio: American Society of Tropical Medicine and Hygiene 527 528 10.4269/ajtmh.1978.27.372 646031

[B11] Shope RE de Andrade AH Bensabath G Causey OR Humphrey PS The epidemiology of EEE WEE, SLE and Turlock viruses, with special reference to birds, in a tropical rain forest near Belem, Brazil Am J Epidemiol 1966 84 467 477 6005906 10.1093/oxfordjournals.aje.a120659

[B12] Peluso RW Richardson JC Talon J Lock M Identification of a set of proteins (C' and C) encoded by the bicistronic P gene of the Indiana serotype of vesicular stomatitis virus and analysis of their effect on transcription by the viral RNA polymerase Virology 1996 218 335 342 10.1006/viro.1996.0202 8610460

[B13] Spiropoulou CF Nichol ST A small highly basic protein is encoded in overlapping frame within the P gene of vesicular stomatitis virus J Virol 1993 67 3103 3110 8388490 10.1128/jvi.67.6.3103-3110.1993 PMC237647

